# Ovariotomy

**Published:** 1873-07

**Authors:** 


### Ovariotomy.

In the continuation of his paper on this subject, an abstract of of the first portion of which appeared in the April number of this journal, Dr. Sims report two cases in detail treated by drainage, the tube being inserted at the time of the operation and describes the different sort of drainage-tubes he has used experimentally.

In commenting on these cases he says : "Septicæmia under certain circumstances will kill just as easily in five or six hours, as under others in five or six days." He states as the result of his experience in the Franco-Prussian war, that the seven cases of wounds of the abdomen above the pelvis which he saw, all died, most of them within twenty-four hours, from septicæmia; but if the pelvic peritoneal cavity was opened by the projectile even although both the bladder and rectum were wounded, they recovered; for in these cases there was a direct outlet or drainage of the reddish or septic serum, which is always the cause of death when retained.

"Wounds of the abdomen, properly speaking, were universally fatal, because the septic fluids could not escape, but gravitating to the lower part of the cavity, were there retained and absorbed, thus producing death. Pleuritis was formerly occasionally fatal, but nowadays no one dies of it. It terminates sometimes by an abundant effusion that may kill by suffocation, and again in the exudation of a pyæmic fluid that kills by blood-poisoning. But in this enlightened day death never occurs in this way, because the effusions, whether benign or pyæmic, are promptly evacuated and life is saved. And so it will be in diseases and wounds of the peritoneum. The time will assuredly arrive when peritonitis, so called, will not kill, because we will learn that the effusions in the peritoneal may be as safely evacuated as those of the pleural cavity ; that the danger will consist not in opening the peritoneal cavity, but in keeping it closing with its retained fluids to poison the blood and take the life of the poor sufferer. The time will come when gunshot and other wounds of the abdomen, and perforations of the intestine, will be treated by opening the peritoneal cavity, and washing out or draining off the septic fluids that would otherwise poison the blood; for death in all these cases is produced by the same causes, in precisely the same way, and they will require the same plan of treatment."—*N. Y. Med. Journal*, April, 1873.—*The Obstetrical Journal.*

